# Smart exercise in pediatric oncology: enhancing executive functions through cognitively challenging physical activity– a non-randomized controlled trial

**DOI:** 10.1186/s12885-025-15303-5

**Published:** 2025-12-05

**Authors:** Ann Christin Schneider, Lisa Hillebrecht, Lars Rehbein, Julia Schmid, Sabine Pallivathukal, Nicolas von der Weid, Rhoikos Furtwängler, Regula Everts Brekenfeld, Jeanette Greiner, Christina Schindera, Eva Katharina Brack, Valentin Benzing

**Affiliations:** 1 Institute of Sport Science, University of Bern, Bern, Switzerland; 2 Department of Pediatric Hematology/Oncology, University Hospital Inselspital Bern, Bern, Switzerland; 3 Department of Pediatric Hematology/Oncology, University Children’s Hospital Basel, Basel, Switzerland; 4 Division of Neuropediatrics, Development and Rehabilitation, Department of Pediatrics, Inselspital Bern, Bern, Switzerland; 5 Department of Pediatric Haematology/Oncology, Cantonal Hospital Aarau, Aarau, Switzerland; 6 Childhood Cancer Research Group, Institute of Social and Preventive Medicine, University of Bern, Bern, Switzerland

**Keywords:** Children with cancer, Exercise, Cognition, Intervention

## Abstract

**Introduction:**

Children and adolescents, diagnosed with cancer, frequently develop physical and cognitive impairments. Hence, cancer and its treatments contribute to reduced physical activity (PA) and cognitive impairments, particularly in executive functions (EFs). Research indicates that PA in school children improves EFs, with cognitively challenging PA offering potential additional benefits. The aim of this study is to investigate the effects of cognitively challenging PA during acute cancer care on cognitive and physical performance, as well as mental health.

**Methods:**

This prospective, two-arm, non-randomized, multicenter controlled study will take place at four pediatric oncology centers in Switzerland. In the intervention group the effect of cognitively challenging PA (*n* = 35) will be compared with standard care plus PA recommendations in the control group (*n* = 35) in newly diagnosed pediatric patients with cancer aged 6-17.99 years. The twelve-week cognitively challenging PA intervention consists of three-weekly 45-minute individualized and supervised sessions incorporating cognitive elements. Assessments of EFs, motor abilities, cardiovascular health, health-related quality of life, fatigue, and physical and psychosocial functioning will be conducted at baseline, six weeks, twelve weeks, and a six month follow-up. All participants in the intervention and control group will receive PA recommendations during the intervention period and an offer for post-therapy PA counseling.

**Discussion:**

Childhood is a crucial period for brain and motor development, rendering young cancer patients especially vulnerable to cognitive and physical impairments from the disease and its treatment. This study is the first to implement cognitively challenging PA tailored to pediatric cancer patients with the aim to enhance EFs by activating brain networks responsible for higher-order processes, physical performance and mental health. The findings will provide insights into the role of cognitively challenging PA and explore its integration into standard care to improve quality of life for childhood cancer survivors.

**Trial registration:**

ClinicalTrials.gov (NCT06839794) and German Clinical Trial Register (DRKS00036573)

## Background

A cancer diagnosis and its treatment in children and adolescents frequently leads to physical impairments such as decline in fitness, muscle loss, and frailty [1, 2]. Over the past decades, the 10-year overall survival rate in high-income countries has significantly improved to 80% [3]. However, this improvement has shifted attention toward the potential long-term impairments associated with the disease, its treatment and the manifested reduced physical activity (PA) [4–6]. 70% of all childhood cancer survivors (CCSs) develop a chronic health condition within 30 years of cancer diagnosis [7]. The most common late effects of CCSs include cardiovascular diseases, hearing loss, renal function impairment, endocrine dysfunctions, hypothyroidism, infertility, as well as neurological and cognitive impairments [8].

Cognitive late effects, including impairments in attention, memory, and processing speed affect around 35–60% of CCSs [9]. Risk factors for cognitive impairments in pediatric cancer patients include high cranial radiation doses, large irradiated brain volume, younger age at diagnosis, and central nervous system (CNS) surgery [9]. These impairments affect particularly higher-order cognitive domains, such as executive functions (EFs) [9], which are required for performing and monitoring goal-oriented, adaptive, and flexible behavior [10]. EFs are essential in unfamiliar, complex, and demanding environments and conditions [10]. One EF model suggests three core processes of EFs (a) inhibition, (b) working memory, and (c) cognitive flexibility, that are important for inhibiting predominant responses and controlling selective attention, for processing and retaining information; and shifting between mental sets and tasks [11]. Additionally, EFs predict a wide range of developmental outcomes (self-control, frustration tolerance, physical health) and academic achievement in children [12, 13].

Apart from these long-term effects, hospitalized children and adolescents often engage in less PA than their healthy peers, spending more time sedentary in bed [5, 14, 15]. Chemotherapy exerts a considerable impact on patients’ activity levels due to its various limiting side effects such as nausea, fatigue, anemia, muscle weakness, and peripheral neuropathy [6, 16]. Consequently, long hospital stays and bed restriction may lead to excessive rest and physical deconditioning [17]. Research shows that, sedentary behavior persists into survivorship [18]. This could contribute to the impaired motor abilities observed in CCSs compared to healthy controls, which are correlated with a lower physical self-concept. Both aspects are linked to health-related quality of life (HrQoL) in CCSs [19]. Taken together, disease, treatment and reduced PA level result in long-term impacts across psychosocial, physical, and cognitive domains [19–23]. Given the significant long-term physical as well as cognitive side effects, there is a pressing need for prevention and early treatment strategies [23].

In recent years, a growing body of evidence highlights the positive impact of PA during acute therapy and PA counseling in aftercare [6, 24–30]. PA has been shown to enhance fitness, sleep, balance, motor abilities, body composition, mobility, HrQoL, and fatigue in children and adolescents with cancer in acute therapy or aftercare [6, 25, 31, 32]. However, empirical evidence is limited when it comes to the effects of PA on cognitive performance during acute therapy. Initial evidence shows that cognitive cancer-related impairment in children after acute cancer care, can be reduced through PA, yielding better cognitive performance than standard care or no intervention [33–35]. CCSs, treated with cranial radiotherapy for a brain tumor performing aerobic exercises, showed alterations in white matter integrity and hippocampal volume and improved reaction time compared to no exercise training in a crossover design [34].

However, specificity of PA interventions is considered an important factor for the benefits of PA for cognitive performance [36, 37]. In recent years, increasing attention has been given to the concept of *cognitive challenge*, defined as the degree of attentional resources and cognitive effort required to master complex tasks [36, 38]. Systematic reviews and meta-analyses indicate that cognitively challenging PA plays a crucial role in enhancing EFs in children and adolescents [39–43]. This is explained by the cognitive stimulation hypothesis [44] suggesting that cognitively challenging PA enhances cognition by activating brain networks involved in higher-order processes [44–46]. Cognitively challenging PA includes for example learning a sequence of movements, where positive feedback can even boost benefits for EFs [38, 47]. Regarding the pediatric oncology field, a feasibility study has explored the effects of sensorimotor training combined with cognitive tasks in pediatric survivors of posterior fossa tumor with positive effects on visual-motor performance [48]. To our knowledge, during acute cancer care, only one study has investigated the effects of combining physical and attentional activities on standardized cognitive performance [49]. However, this study did not include a control group without PA [49]. Showing that, research continues to explore the combination of PA and cognitive tasks to evaluate their potential for more effectively mitigating cognitive impairments [48, 49].

Taken together research is needed on the effects of cognitively challenging PA on both cognitive and physical performance in children and adolescents with cancer undergoing acute treatment. In the present study, we aim to address this while also ensuring that participants continue to receive appropriate support following the PA counseling offered—an element recognized as essential in pediatric oncology care [24]. Additionally, our approach aligns with the recommendations of the International Late Effects of Childhood Cancer Guideline Harmonization Group, which highlights the importance of targeting cognitive functioning and HrQoL in this vulnerable population [33].

### Objectives and hypotheses

The primary objective of this study is to investigate whether a twelve-week cognitively challenging PA intervention in children and adolescents with cancer has a positive effect on their EFs.

Secondary objectives are to investigate whether the twelve-week cognitively challenging PA program has a positive effect on cardiovascular health, motor abilities, body composition, psychosocial functioning, HrQoL and fatigue. Additionally, feasibility, safety, and adverse events of our PA program will be assessed. A PA counseling in maintenance therapy or aftercare will be offered to all participants, and its feasibility will be explored using qualitative interviews.

## Methods

The study is an investigator-initiated, two-phase, non-randomized multicentre controlled trial. During the first phase, the intervention will commence at the Inselspital Bern, while the University Children’s Hospitals Basel and Zurich, as well as the Children’s Hospital Aarau will serve as control sites. In the second phase, after 1.5 years, the intervention will be implemented in Basel, Zurich and Aarau, with Bern serving as the control. Due to ethical considerations, randomization of participants within a single center was not feasible; therefore, the control group consists of participants from other centers.

### Participants

In total, a minimum of 70 participants (for details, see sample size calculations), will be included, with 35 assigned to the intervention (IG) and 35 to the control group (CG).

Children and adolescents aged 6–17 years with a diagnosis of any type of cancer requiring chemo- and/or radiotherapy, expected to last a minimum of at least six weeks at the time of recruitment, and/or CNS surgery, will be included.

Participants will be excluded if they have cognitive and physical disabilities (e.g. down syndrome, cerebral palsy), that prevent participation in the intervention. Additionally, the inability to follow the procedures of the study, e.g. due to language problems, is an exclusion criterion.

### Recruitment

Patients will be screened for eligibility according to the inclusion and exclusion criteria and recruited by a study investigator or treating physician. No additional screening procedures are required beyond verifying inclusion and exclusion criteria. As this is a two-phase study, enrolment into the IG and CG is timely and locally determined.

No monetary compensation will be provided to study participants. However, small thank-you gifts and vouchers will be given as a token of appreciation for participation.

### Intervention for IG

The intervention is based on the “S2k Guideline: Promotion of Exercise and Exercise Therapy in Pediatric Oncology”, the international Pediatric Oncology Exercise Guidelines [1, 50] and findings and experience from our previous cognitively challenging PA intervention studies [51–54]. The intervention consists of cognitively challenging PA designed for children and adolescents undergoing acute cancer treatment (Table 1).

**Table 1:** Design of the cognitively challenging PA session during acute therapy

Elements of PA session	Time (minutes)	Content
Welcome	3	State of health and overview of the PA session
Warm-up	5	Opening ritual or game
Cognitively challenging PA	5	PA incorporating cognitive challenges focusing on EFs
Individualized PA	5–30	Strength, endurance, coordination, flexibility, relaxation, balance, cognition
Cool-down	3	Closing ritual or game, e.g. short breath exercises
Reflection and goodbye	2	PA diary

*EFs* Executive functions, *PA* Physical activity

The entire intervention is designed to be playful, and physically, and cognitively challenging. The intervention will be conducted over a twelve-week period, during which the participants will engage in supervised training sessions of 45 minutes, three times a week. The timing, frequency and intensity will be adjusted to accommodate each child’s and adolescent’s current health and fitness status as well as individual abilities. This adaptive approach prevents overexertion and ensures that all participants can actively engage while being optimally challenged. Each PA session follows a structured format: 1. warm-up phase, 2. cognitively challenging PA task, 3. multimodal PA elements designed to train functional mobility, cardiopulmonary fitness, and muscle strength [1]. Examples of the PA elements are listed below:

– Warm-up: PA games, as holding a balloon high
– Cognitively challenging PA: Each exercise session incorporates cognitively challenging PA, targeting EFs such as inhibition, cognitive flexibility, and working memory. A representative example is the stop-dance activity, where participants must abruptly stop moving when the music ceases. An extension is that the children are required to execute specific stopping actions in response to predetermined commands.
– Strength: Strengthening exercises utilizing small equipment (e.g., weights, books, mini-bands), such as performing glute bridges with mini-bands around the legs.
– Endurance: Activities including jumping, running, and dancing, integrated into endurance-based games such as walking memory.

At the end of each session, participants complete an exercise diary, in which the training session is summarized. To minimize drop-out rates after temporary or permanent discharge from hospital, a similar online program will be offered to the participants in the IG. This digital alternative will allow them to maintain engagement in PA, ensuring continuity of the intervention even outside the hospital setting.

### Intervention for CG and IG

#### PA recommendations

All children and adolescents will receive general PA recommendations (based on the above-mentioned guidelines) following the baseline measurement. After the twelve-week measurement (T2), participant will receive individualized PA recommendations in the form of a personalized letter. This letter provides information on the patient’s performance during a motor ability test (Moon test; see Measurements) and offers recommendations for improving motor abilities through age-appropriate PA tasks.

#### PA counseling

In addition, to support long-term PA engagement, all participants in maintenance therapy and aftercare will receive a PA counseling. This will consist of two to four guided counseling sessions aimed to assist reintegrate children and adolescents into local sports club, school sport and leisure-time activities. The counseling sessions will take place, when maintenance therapy starts. During the counseling, the patient’s individual motives for exercise and sport will be addressed, and matching activities will be identified [55, 56]. This personalized approach allows for determining feasible and beneficial PA for the patient [55].

### Study procedure and measurement time points

**Fig. 1:**
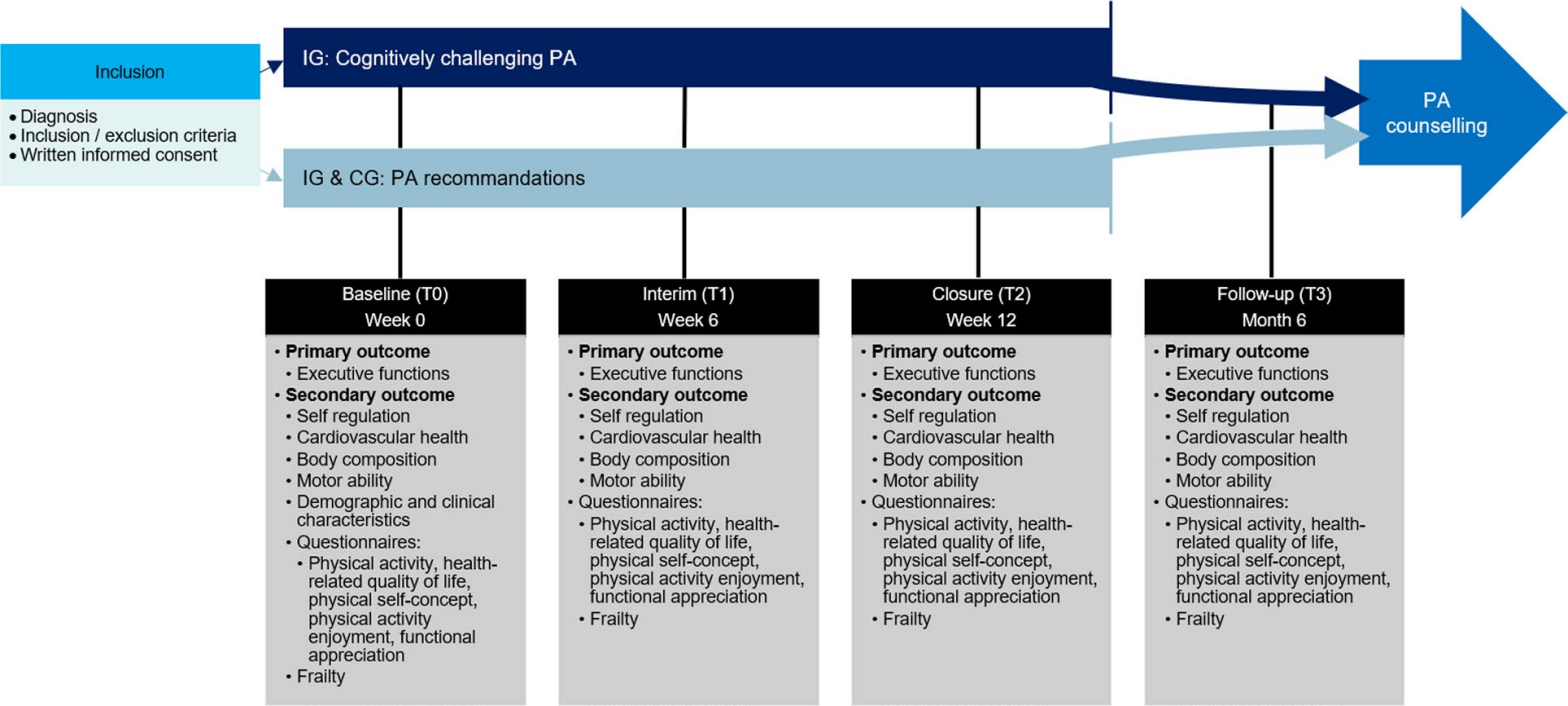
Study design and measurements. CG = Control group, IG = Intervention group, PA = Physical activity

Participants will be enrolled in the study within the first three weeks after their cancer diagnosis. Subsequently to study inclusion, the baseline measurement will be conducted (T0). Afterwards, children in the IG will participate in a twelve-week cognitively challenging PA program. After six weeks, an interim measurement (T1) is carried out for both groups and again after twelve weeks at the end of the intervention (T2). To record possible long-term effects of the intervention, a follow-up measurement is carried out after six months (T3). At each of these four predefined and above-described measurement time points, all assessments will be conducted during a routine outpatient visit and/or during hospitalization (Fig. 1).

### Outcome measures

To avoid carry over effects from physical to cognitive assessments, physical performance assessments will be placed at the end. For each participant, the assessment will be as follows: Body composition, assessment of EFs, cardiovascular measurements except endurance measurement, motor ability related measurements and the 6 min-Walking-Test at the end. The questionnaires are distributed last.

#### Executive Functions (EFs)

The following table provides a summary of three cognitive function tests, detailing their primary purpose, task procedure, duration, target age group, and key outcome measures. These tests assess the three core EFs, including inhibition, cognitive flexibility and working memory. They have been validated across different age ranges. (Table 2). 

**Table 2:** Summary of the primary outcome measurements (EFs)

Test Name	Cognitive Function Assessed	Task Description	Duration (minutes)	Age Range (years)	Outcome Measures	Reliability	Ref.
Hearts and Flowers Task	Inhibition and cognitive flexibility	Participants sit facing a screen and respond by pressing a button on the corresponding side when a heart stimulus appears and on the opposite side when a flower stimulus is displayed. Three blocks: congruent (hearts), incongruent (flowers), and mixed (both).	5	4–45	Reaction time, accuracy, composite score	Cronbach’s alpha = 0.83 Test-retest reliability: 0.62 for accuracy score 0.72 for reaction time	[10, 57–61]
Fish Flanker Task	Inhibition	Participants sit in front of a screen and respond to the orientation of a central fish while ignoring surrounding fish, which may show in congruent or incongruent directions.	8	6–18	Reaction time, accuracy, composite score	ICC = 0.92	[62, 63]
Digital Corsi Block Task	Visuospatial working memory	Participants replicate sequences of illuminated blocks on a digital screen, with increasing sequence length in case of correct responses.	varies	>7	Block span, total correct sequences, summary score	ICC = 0.93 for children, ICC = 0.76 for adolescents	[64–69]

*ICC* Intraclass correlation coefficient

#### Self-regulation, cardiovascular health, body composition, motor abilities, PA levels, HrQoL and others

The following table summarizes various health assessments used to evaluate self-regulation, cardiovascular health, body composition, motor abilities, PA levels, HrQoL, fatigue, physical self-concept, PA enjoyment, body image and frailty in children and adolescents, particularly in the context of cancer therapy. Each assessment’s focus, methodology, duration, target age group, and key outcome measures are outlined (Table 3).

**Table 3:** Summary of the secondary outcome measures

Secondary Outcomes	Measurement Focus	Methodology	Age Range	Outcome Measure	Reliability and Validity	Ref.
Self-Regulation	Self-Regulation	Head-Toes-Knees-Shoulder Task: Participants follow commands with rule-based changes (e.g., ‘If I touch my foot, you touch your head’), with increasing complexity over time.	3 to 68.55 years	Correct responses	Test-retest reliability (*r* = 0.60–0.66) Construct validity (*r*_*s*_ = 0.46–0.56)	[70–74]
Cardiovascular Health	HRV	Chest belt (EcgMove 4)	Children and adolescents undergoing cancer therapy	HRV		[75–80]
	Aortic stiffness	Mobil-O-Graph		PWV		
	6MWT	6 min walking distance		6MWT distance	Test-retest ICC = 0.944, correlation with VO2max (*r* = 0.61)	
Body Composition	Body fat percentage, skeletal muscle mass	BIA after fasting for eight hours	Children and adolescents undergoing cancer therapy	Body fat percentage, skeletal muscle mass	Utilized in pediatric cancer patients	[81–85]
	Skinfold thickness	Caliper		Triceps skinfold thickness	Validated in pediatric cancer patients	
Motor Abilities	Strength, speed, coordination, flexibility	MOON test with seven items: pin insertion, static stance, reaction time, sit-and-reach, medicine ball throw, stand-and-reach, hand grip strength	4 to 18 years (normative data: 6 to 17 years)	Performance on MOON test	Feasibility established in pediatric cancer patients	[86–89]
	Postural control	Bilateral- (EC), semitandem- (EO) and unilateral (EO) stand	Children and adolescents undergoing cancer therapy (5 to 18 years)	Centre of pressure measured by a force plate (Leonardo, Novotec, Pforzheim, Germany)		
PA Levels	Self-reported PA levels	Questionnaire: BSA	Children and adolescents	Last 4 weeks of sporting activity: 6 items	PA correlation with anaerobic threshold (*r* = 0.27–0.35)	[90–94]
	PA intensity	Questionnaire: GSLTPAQ (abbreviated German-language version)		Light, moderate, and vigorous PA levels: 3 items	Modified version: test-retest reliability of *r* = 0.68	
HrQoL and Fatigue	Physical, emotional, social, and academic functioning	Questionnaire: PedsQL Generic Core (physical, emotional, social, academic)	5 to 18 years	Scores: PedsQL Generic Core: 23 items	Reliability (Cronbach’s α = 0.88–0.93) Construct validity confirmed	[95–97]
	Fatigue	Questionnaire: PedsQL Multidimensional Fatigue Scale		Scores: Multidimensional Fatigue Scale: 18 items	Reliability (Cronbach’s α = 0.89–0.92) Construct validity confirmed	
Physical Self-Concept	Coordination, strength, flexibility, endurance, and the two global scales: physical self-concept and self-esteem	Questionnaire: PSDQ-S for ages 11–17	8 to 17 years	Scores: 22 items	Reliability (Cronbach’s α = 0.79–0.90)	[98–101]
		Questionnaire: PSQC-C for ages 8–12		Scores: 15 items	Reliability (Cronbach’s α = 0.77–0.90)	
PA Enjoyment	Enjoyment of PA	Questionnaire: Short version of PACES-S	11 to 17 years	Scores: 4 items	Test-retest reliability (*r* = 0.76) Internal consistency (Cronbach’s α = 0.87)	[102, 103]
Body Image	Appreciation of body function	Questionnaire: German FAS	Children and adolescents	Scores: 7 items	Reliability (Cronbach’s α = 0.88), Moderate validity (*r* = 0.65, *p* < 0.05)	[104, 105]
Frailty		Series of five measurements: weakness (hand strength), slowness (6MWT), shrinkage (triceps skinfold), exhaustion (PedsQL multidimensional fatigue scale), PA (BSA, GSLTPAQ)	Children and adolscents	Frailty Score		[84]

*BIA* Bioimpedance analysis, *BSA* [Bewegungs- und Sportaktivität Fragebogen] Physical activity and sports questionnaire, *EC* Eyes closed, *EO* Eyes open, *FAS* Functionality Appreciation Scale, *GSLTPAQ* Godin-Shephard Leisure-Time Physical Activity Questionnaire, *HRV* Heart rate variability, *HrQoL* Health-related quality of life, *PA* Physical activity, *PACES-S* Physical Activity Enjoyment Scale – short version, *PSQC-C* Physical Self-Description Questionnaire for Children, *PSDQ-S* Physical Self-Description Questionnaire – short version, *PWV* Pulse-wave velocity, *Ref* Reference, *6MWT* 6 Minute Walking Test

### Other measurements and assessments

#### Baseline characteristics

Baseline characteristics (age, sex, diagnosis, height, weight) of participants will be recorded at the beginning. These are retrieved via the clinic’s internal software program. In addition, the socio-economic status is queried using the Family Affluence Scale [106].

#### Chemotherapy intensity and therapy protocol

Chemotherapy intensity, classified into four levels based on the duration of severe neutropenia will be assigned to each participant using their clinical records [107, 108]. Furthermore, the patient’s therapy protocol will be documented.

#### Feasibility

For quality assurance purposes, feasibility and implementation outcomes will be assessed using a self-developed questionnaire. Further, a subsample of participants will be qualitatively interviewed using semi-structured interviews, which will be transcribed and analyzed with qualitative content analysis.

### Statistical analysis

#### Sample size calculation

Two sample size calculations were performed for the primary outcomes: one encompassing both inhibition and cognitive flexibility and another for working memory. The calculation for the former is presented below, as it yielded the larger sample size needed, which was therefore selected for the study.

The sample size calculation was done using G*Power 3: Repeated measures ANOVA with four measurement points and two groups (within-between interaction), a statistical power of 80%, a small effect size (Cohen’s *f* = 0.105) [54], an α = 0.05 and a retest correlation of 0.8. This results in a minimum sample size of *N* = 52 in total, i.e. 26 subjects per group. To compensate for losses and drop-outs, we set the minimal sample size to *N* = 70.

#### Statistical analysis plan

Study sample characteristics will be shown separately as mean values (x̄) with its standard deviation (±) for each group regarding continuous variables. For categorical variables percentages and absolute numbers will be used. Normal distribution will be analyzed by the Shapiro-Wilk test and graphical verification of the Gaussian distribution. For all analyses, the significance level will be set at *p* < 0.05 and effect sizes will be provided.

*Preliminary analyses* will be performed to ensure that characteristics of the study participants (e.g., age, sex, diagnosis, weight, height) are comparable between the IG and CG using independent *t-*tests (continuous variables) or chi-squared tests (categorical variables). For feasibility analysis, descriptive statistics will be used.

The *main analyses* for the primary and secondary outcomes are repeated measures ANOVAs (for normal distributed data) and the Friedman test (for non-normally distributed data), where the “between-group” factor describes the IG vs. CG and the “within-group” factor is “timepoint” (T0, T1, T2, T3). If linear mixed models converge (despite small sample size), they will be used instead of repeated measures ANOVAs. These models will be specified to adjust for potential baseline imbalances [109]. They include the main effect of time and the group-time interaction. As the period between the data collection waves most probably will not be equal for all participants, as a time variable, the individual time interval in days will be used to increase the precision with which a child’s growth trajectory is measured. For each dependent variable, the optimal model will be determined using likelihood-ratio tests for nested models and Akaike information criterion for non-nested models. For these comparisons a random intercept model will be compared to a fixed effects model and a model including random intercepts and fixed effects. The model with the best fit will subsequently be chosen.

For *supplementary analyses*, we will perform the same analyses for different age groups (6–11.99 and 12–17.99 years), cancer types (e.g. leukemia, brain tumors) and chemotherapy severity levels with sufficient sample size (*n* > 20). In addition, baseline performance will be correlated with gain scores using Pearson correlations (for normally distributed data) or Spearman correlations (for non-normally distributed data) for outcomes that show significant group × time interactions. To explore associated changes in certain variables, changes in motor abilities will be correlated with changes in core EFs and psychosocial functioning.

Additionally, for the intention-to-treat (ITT) analyses, we will perform two per protocol analyses. In the first per protocol analyses, only participants will be included, who did at least 80% of the PA sessions, in the second per protocol analyses participants will be included, if they completed 60% of training sessions. For all statistical analyses, the statistical program R and SPSS will be used.

#### Missing data

Dropouts will be documented with detailed information regarding their causes. Dropouts will not be replaced to comply with the ITT principle. Depending on the amount of missing data, it will be handled using single (< 5% missing data) or multiple imputation (≥ 5% missing data) methods or in the case of linear mixed models with maximum likelihood estimation.

#### Data management

RedCap will be used for data storage hosted by the Clinical Trials Unit Bern. For each participant, an electronic case report form is maintained within RedCap. This means that all digital data will be stored there. Data initially recorded in paper format (storage in locked files) will be digitalized and transferred to RedCap. Subject confidentiality will be ensured by utilizing subject identification coding numbers.

#### Data monitoring

Data monitoring will be conducted regularly, including routine verification of data quality performed by the principal investigator or his designees. All severe adverse events will be documented and reported immediately to the principal investigator. Furthermore, the Swiss Ethics Committee reserves the right to carry out audits and inspections at any time with full access to the data.

## Discussion

This study protocol outlines the design and methodology to investigate the cognitive and physical effects of cognitively challenging PA in pediatric cancer patients. Research suggests, that engaging in cognitively challenging PA in a playful setting has a beneficial effect on cognition, particularly in healthy children [53, 110]. This is because such exercises activate specific brain networks thereby enhancing EFs [36, 111]. Therefore, this approach could possibly serve as a preventive strategy for cognitive and physical impairments in children and adolescents undergoing cancer treatment [5, 9, 45, 49]. The intervention is designed based on the “S2k Guideline: Promotion of Exercise and Exercise Therapy in Pediatric Oncology” and the international Pediatric Oncology Exercise Guidelines [1, 50]. Additionally, it incorporates evidence from previous research studies and practical insights gained during cognitive training and PA interventions to optimize its effectiveness [51–54]. Finally, combining PA and cognitive tasks simultaneously can improve cognitive performance and encourages the children to remain active [36, 37, 112].

The main strength of this study is its uniquely designed approach for children and adolescents with cancer. Since the PA intervention offers varying levels of difficulty for the physical and cognitive tasks it ensures inclusivity. Through supervision, and knowledge of the underlying disease, the physical and cognitive intensity levels can be adjusted individually to avoid overload and to provide optimally cognitively challenging PA. Furthermore, integrating supervised PA during acute cancer care with structured PA counseling for the post-acute phase facilitates the promotion of sustained long-term effects. The study addresses a difficult-to-reach patient population, as participants alternate between inpatient and outpatient visits. We mitigate this and so reduce dropout by offering online exercise sessions, reaching patients at home.

A key limitation of the study is the heterogeneity of the patient population with different cancer types, age, treatment methods and duration. Moreover, randomization is not feasible, as it is ethically unjustifiable to withhold the PA intervention from eligible children and adolescents in the same clinic. Furthermore, the patients’ state of health may lead to low adherence to PA and missing data. Additionally, blinding of the study staff is not possible due to the study design of the intervention group assignments.

## Conclusion

Childhood is the most critical period for brain development, making pediatric cancer patients particularly vulnerable to neurocognitive impairments, as well as physical disabilities due to the cancer and its therapy [1, 5, 113, 114]. More research needs to be devoted to children with cancer and their development since cognitive and physical abilities, growth, and reintegration into school are essential for their future life [20, 25]. It will provide new insights into the role of cognitively challenging PA as an intervention to enhance cognitive and physical performance in pediatric cancer patients. Adopting a more applied perspective, this study could explore the integration of a specialized PA program into routine clinical care as a potential approach to address cognitive impairments and in general improve the quality of life for CCSs.

## Data Availability

No datasets were generated or analysed during the current study.

## Abbreviations

CCS: Childhood cancer survivor
CG: Control group
CNS: Central nervous system
EFs: Executive functions
HrQoL: Health-related quality of life
IG: Intervention group
ITT: Intention-to-treat
PA: Physical activity
